# Diatomaceous earth/zinc oxide micro-composite assisted antibiotics in fungal therapy

**DOI:** 10.1186/s40580-021-00283-6

**Published:** 2021-10-25

**Authors:** Huifang Liu, Zhen Qiao, Yoon Ok Jang, Myoung Gyu Kim, Qingshuang Zou, Hyo Joo Lee, Bonhan Koo, Sung-Han Kim, Kyusik Yun, Hyun-Soo Kim, Yong Shin

**Affiliations:** 1 Department of Biotechnology, College of Life Science and Biotechnology, Yonsei University, Seoul, 03722 Republic of Korea; 2 Department of Infectious Diseases, Asan Medical Center, University of Ulsan College of Medicine, 88 Olympicro-43gil, Songpa-gu, Seoul, 05505 Republic of Korea; 3 Department of Bionanotechnology, Gachon University, Gyeonggi-do Seongnam, 13120 Republic of Korea; 4 INFUSIONTECH, Gyeonggi-do 427 beon‐gil, Dongan‐gu, Anyang‐si 14059, Republic of Korea

**Keywords:** Antifungal therapy, Antibiotic efficacy, Persistent therapy, Synergistic effect, Biocompatibility

## Abstract

As the second wave of COVID-19 hits South Asia, an increasing deadly complication ‘fungal infections (such as Mycosis, Candida and Aspergillus) outbreak’ has been raised concern about the insufficient technologies and medicals for its diagnosis and therapy. Biosilica based nano-therapy can be used for therapeutic efficacy, yet their direct role as antibiotic agent with biocompatibility and stability remains unclear. Here, we report that a diatomaceous earth (DE) framework semiconductor composite conjugated DE and in-house synthesized zinc oxide (DE-ZnO), as an antibiotic agent for the enhancement of antibiotic efficacy and persistence. We found that the DE-ZnO composite had enhanced antibiotic activity against fungi (*A. fumigatus*) and Gram-negative bacteria (*E. coli, S. enterica*). The DE-ZnO composite provides enhancing large surface areas for enhancement of target pathogen binding affinity, as well as produces active ions including reactive oxygen species and metal ion for breaking the cellular network of fungi and Gram-negative bacteria. Additionally, the toxicity of DE-ZnO with 3 time less amount of dosage is 6 times lower than the commercial SiO_2_-ZnO. Finally, a synergistic effect of DE-ZnO and existing antifungal agents (Itraconazole and Amphotericin B) showed a better antifungal activity, which could be reduced the side effects due to the antifungal agents overdose, than a single antibiotic agent use. We envision that this DE-ZnO composite can be used to enhance antibiotic activity and its persistence, with less-toxicity, biocompatibility and high stability against fungi and Gram-negative bacteria which could be a valuable candidate in medical science and industrial engineering.

**Supplementary Information:**

The online version contains supplementary material available at 10.1186/s40580-021-00283-6.

## Introduction

The recent BBC report showing that those who have been cared in intensive care unit (ICU) of severe COVID-19 are vulnerable to deadly infections [1–4]. In comparison with the death rate of COVID-19 (about 2.1%, calculate by 28th June, 2021), the high fatality rate of these Invasive Aspergillosis infection (IAI, such as mycosis: 70%, candida: 25%, aspergillus: 30 ~ 90%) has sent people into another serious pandemic [5, 6]. Nowadays, resistance to and the toxicity of antibiotics are a concern due to the rising incidence of microbial infections with serious side effects in world healthcare settings [7, 8]. Particularly, infections caused by multidrug-resistant bacteria (super bugs), fungi, Gram-negative bacteria, or Methicillin-resistant *S. aures* (MRSA), remain difficulty to treat due to their resistance to multiple antibiotics [9]. In addition to fungal infection, these conditions debilitate the human immune system, which leads to increased mortality from fungal infection among cancer patients, transplant recipient and those patients with damaged blood vessels and other airway wall by COVID-19 [10, 11]. The fungal (in spore or conidium) with independent survive ability are ubiquity which have challenged the environmental engineering and industrial production on prevention for building a world of health and warmth [12, 13].

Invasive Aspergillosis infection (IAI) is a more frequent direct cause of death than mortality from other diseases [14, 15]. Especially, the treatment of IAI caused by *Aspergillus fumigatus, Candida albicans,* and *C. glabrata* are constrained due to the toxicity of the agents, drug resistance, and low efficacy. *Aspergillus* species kill up to 80% of infected patients [16, 17]. Therefore, the ubiquitous and harmful *Aspergillus*, which can be found in the dust in your home (moldy walls, molds on foods), can induce lesions without clear symptoms and has been set as an experimental subject [18, 19]. According to the statistical reports, around 75% of antibiotics were consumed not by humans, but by livestock, and many developed countries have no restrictions on this use [16, 20]. Generally, drugs with antifungal activity that are used to treat patients with IAI are azoles, polyenes, and echinocandins [17]. Among these drugs, amphotericin B is one of polyenes, used to break the membrane of fungi by binding with ergosterol, which subsequently leads to cell death. Itraconazole is a tolerated azole antifungal drug, affecting the cell membrane directly or after its metabolism [21]. However, the clinical use of these drugs is extremely limited since they have serious side effects, such as nausea, diarrhea, abdominal pain, rash, headache, and organ damage due to their high toxicity, insolubility, and sensitivity to pH [14, 21]. Hence, there is an urgent demand to find better solution, which could have enhanced efficacy and persistence with relatively less toxicity [22].

To address these challenges, numerous nanotechnologies based on nanomaterials have emerged for effective therapy in the field of the nano-medicines [23]. The emerging nanomaterials exhibit unique physical, chemical, and biological properties that are widely studied in various clinical applications [24]. However, many side effects have been noted when moving these technologies from the bench to the bedside due to insufficient effective and timely infection eradication, and toxicity against the surrounding normal cells [11, 25]. Therefore, the development of non-toxic composites that substitute for or assist the antibiotics, which can provide a concrete direction for further clinical use in the medical therapy market, is essential. Recently, biosilica or optimized semiconductors have attracted increasing interest for producing essential elements for the human body [26, 27]. They also show biocompatibility, multifunctionality, and extensive resources. However, biosilica based nano-therapy can be used for therapeutic efficacy, yet their direct role as antibiotic agent with biocompatibility and stability remains unclear. Among the candidate materials, zinc oxide (ZnO) and diatomaceous earth (DE) are listed as safe substances for various applications such as medicines, cosmetics, animals and industries [28, 29]. Zinc oxide (ZnO) nanomaterials have been widely applied in electronics, photo-electronics, antibacterial effect, sensors, and photocatalysts with excellent chemical and conductivity properties [30, 31]. Especially, the dissolution to ionic zinc (Zn^2+^) and particle-induced reactive oxygen species (ROS) from ZnO have represented the primary modes of action for antibacterial purposes [2, 32]. ZnO is intriguing as a candidate drug due to its properties of antibacterial activity, but the complete persistence and efficacy of the antibiotic action of ZnO remains unexplored. DE is being developed as a possible carrier to increase drug loading due to the nano-porous structure of its surface [33]. However, the widespread applications of DE are still limited because of its dosage-dependent toxicity and crystal structure-size effect. Therefore, further study into the optimal utilization and facile-green chemical methods of the candidate materials with clear mechanisms of action are desirable for further clinic therapy applications.

**Scheme 1:**
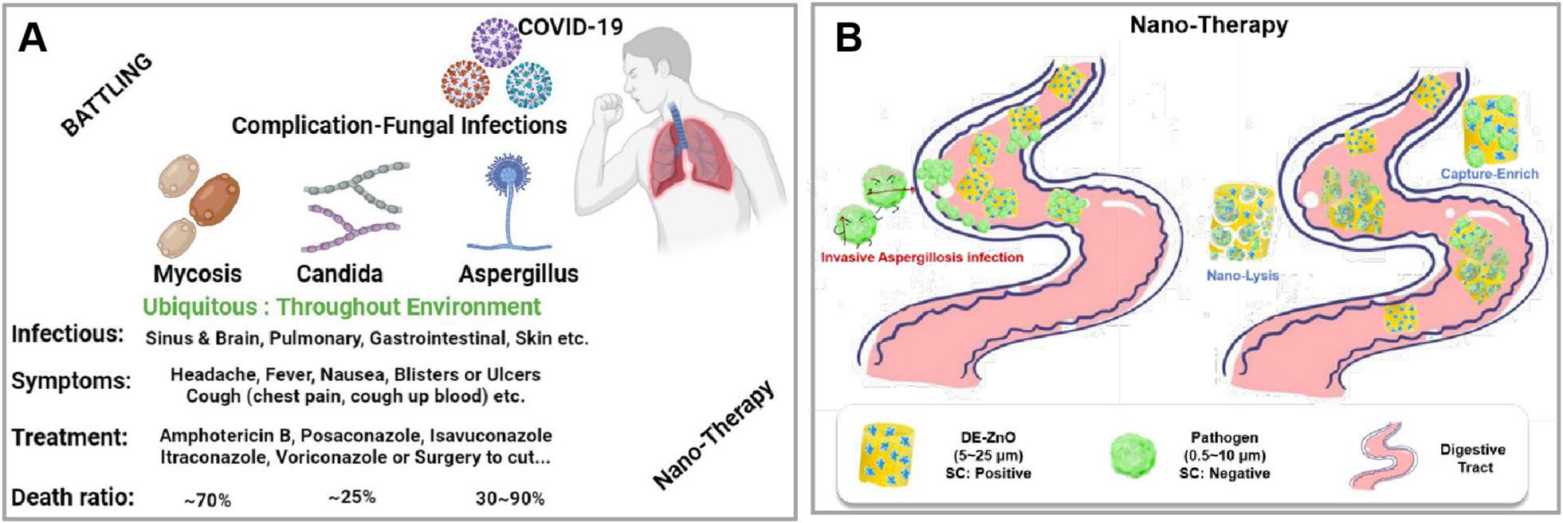
Can nano-therapy help to win the (medical science) battling? **A** Challenges in the therapy of pandemic: complication-fungal infections. **B** Our experimental non-toxic composite with enhanced antibiotic efficacy and persistence against pathogen

Can nano-therapy help to win the (medical science) battling? (Scheme 1A) Here, we report a novel non-toxic composite with enhanced antibiotic efficacy and persistence against fungi and Gram-negative bacteria (Scheme 1B). The non-toxic composites are fabricated with in-house synthesized ZnO and biocompatible frustules DE, called DE-ZnO composites. Toxicity and persistence of antibiotics are important issues for reducing the side effects in patients who suffer from various infectious diseases. Thus, we conducted phenotypic and genetic analysis for both in vitro and in vivo testing of DE-ZnO as an antibiotic agent with negligible toxicity, not as a carrier for drug delivery. Through specific attention to its mechanism of action, we showed that DE-ZnO breaks fungal and bacterial cellular networks by its physical and chemical properties, such as the shape of DE-ZnO and high levels of production of reactive oxygen species (ROS). In addition, we demonstrated that DE-ZnO has a long persistence of its antibiotic effect against fungal (*A. fumigatus*) and Gram-negative bacterial (*E. coli and S. enterica*) infections. Furthermore, we showed DE-ZnO has an antifungal synergistic effect against *A. fumigatus* when used in combination with amphotericin B and itraconazole. Therefore, we believe that the ability of DE-ZnO to enhance the efficacy and persistence with non-toxicity indicates it could be useful as a possible antibiotic agent, as well as an enabler for combination therapy with existing drugs in various antimicrobial applications.

## Methods

### Chemicals and reagents

All reagents were of analytical grade and were used without further purification. Zinc nitrate hexahydrate (ZnNO_3_·6H_2_O, 98%), ammonium hydroxide solution (28% NH_3_ in H_2_O, 99.99% trace metals basis), biocompatible DE powder (Cat. No. D5509), 2′,7′-dichlorofluorescein (Lot # BCBZ6854), zinc oxide nanoparticles (Cat. No. 721077-100G), amphotericin B from *Streptomyces* sp. (Cat. No. A2411-250MG), Itraconazole (Cat. No. I6657-100MG), and 2′,7′-Dichlorofluorescein (DCF) (Cat. No. BCBZ6854) were purchased from Sigma-Aldrich (St. Louis, MO, USA). Hexadecyltrimethylammonium bromide (C_19_H_42_BrN, > 98%, CTAB) was purchased from Tokyo Chemical Industry Co., Ltd. (Tokyo, Japan). Dulbecco’s Modified Eagle’s Medium (DMEM; Life Technologies, Carlsbad, CA, USA) was used for cell culture. LB medium was used for the bacterial cultures. Sabouraud dextrose agar with chloramphenicol media (Cat. No.: C6781; Lot no: 437412) for the fungi culture was purchased from Santa Maria-USA. Milli-Q water and phosphate-buffered saline (PBS, 10× , pH 7.4) were used in all experiments.

### Biological samples

(a) L929 (mouse C_3_H/An connective tissue) and eukaryotic cells (HCT-116 colorectal cancer cells) were maintained in culture dishes with high-glucose DMEM supplemented with 10% fetal bovine serum (FBS) at 37 °C in a 5% CO_2_ atmosphere. (b) The prokaryotic species *Escherichia coli* (*E. coli*, ATCC25922) and *Salmonella enterica* (*S. enterica*, ATCC14028) were inoculated into either nutrient broth medium or LB medium and were incubated overnight at 37 °C with shaking. (c) *Aspergillus fumigatus* (*A. fumigatus*, ATCC36607) were grown in Sabouraud dextrose agar at 25 °C for 5 days. After culturing, the Aspergillus fungi were resuspended in PBS and quantified by using a hemocytometer.

### Preparation of the ZnO NMS and bio-semi-composites (DE-ZnO)

(a) A modified hydrothermal method has been used to synthesize ZnO NMS crystals in alkaline medium. Briefly, 1 mL of 1 M CTAB was added to 98 mL of Milli-Q water in a 250 mL flask, and magnetically stirred at 500 rpm at 90 °C. Then 1 mL of 1 M Zn(NO_3_)_2_·6H_2_O was added and it was stirred for 50 min. Under steady stirring and incubation conditions, 2 mL of ammonium hydroxide solution was added into the reaction mixture drop-wise. As the white precipitates were produced, we transferred the flask into an ice box to stop the reaction. The white precipitates were collected by centrifugation and washed with Milli-Q water three times to wash away the residual ions. Then, the white precipitates were dried in a 56 °C oven overnight. (b) The bio-semi-composites (DE-ZnO) were synthesized by a typical nanoengineering particle surface method. The porous DE acted as a well matrix that supplied a sufficient surface area for the ZnO. Briefly, DE was purified by gravity settling in deionized (DI) water, the uniform DE (0.5 g) was then dissolved in 98 mL of Milli-Q water in a 250 mL flask. Then, 1 M Zn(NO_3_)_2_·6H_2_O and 1 mL of 1 M CTAB were added to the DE solution. Under magnetic stirring at 500 rpm at 90 °C for 50 min, the activated Zn^2+^ diffused and anchored on the surface of the DE by van der Waals forces. Our modified hydrothermal method for synthesis of ZnO NMS has been carried out here, with the ZnO NMS growing in a crystal direction. As we see the color of reaction solution changed from brick-red (DE) to pink-white, we transferred the flask into an ice box to stop the reaction. The produced precipitates were collected by centrifugation and washed with Milli-Q water three times to wash away the residual ions. Furthermore, a gravity settling method was used to wash out the dissociative ZnO NMS. Finally, the precipitates were dried in a 56 °C oven overnight.

### Characterization

The morphology of the ZnO nanomaterials (commercial or synthesized), DE-ZnO, and related biological samples were characterized using Feld Emission Scanning Electron Microscopy (FE-SEM) on a JSM-7500F instrument (JEOL) to confirm the reaction and surface materials. Zeta potentials of the materials were measured using dynamic light scattering (DLS) on a DynaPro NanoStar instrument (Wyatt). Fourier-transform infrared spectroscopy (FTIR) analysis was performed using a JASCO 6300 instrument (JASCO) on bare ZnO-C (~ 100 nm, ~ 5000 nm), (ZnO-S (~ 300 nm), and DE-ZnO to obtain information on the chemical modifications. In addition, the elements present in the composite materials were analyzed using EDX, while UV/visible spectrophotometer measurements were used to determine the composite materials.

### Antibacterial

A suspension of bacteria (*E. coli*, *S. enterica*) was used to evaluate the antibacterial activity of different types of zinc oxides and composites. Before the assay, the bacteria were grown aerobically in LB medium for 16 h at 37 °C in 210 rpm shaking incubator. Then, the bacterial cells were harvested by centrifugation at 2000 rpm for 5 min and the bacterial cells were re-suspended in LB. The bacterial suspension was appropriately diluted (up to 10^6^) and a 100 μL aliquot was transferred to the agar plate. The bacteria were then evenly distributed on the agar plate surface using a plastic rod. After 16 h of incubation, colonies were counted. The counting colony forming units (CFU/mL) were calculated for the original bacteria sample. The bacterial suspension was then adjusted to a fixed concentration of 1 × 10^7^ CFU/mL. Thereafter, different amount of the test nanomaterials were placed into a tube containing 0.1 mL of 1 × 10^7^ CFU/mL bacterial suspension and 2 mL LB medium. After incubating for 16 h at 37 °C shaking at 210 rpm, we measured the absorbance of each sample at OD 600 nm. The bacteria survival rate of each sample was calculated by the equation:

1 $$\begin{aligned} &{\text{Bacteria Survival Rate}} (\% ) \\ &\quad= \frac{\text{OD}_{\text{ZnO}}-\text{OD}_{\text{Negative}}}{\text{OD}_{\text{Positive}}-\text{OD}_{\text{Negative}}} \times 100\% \hfill \\ \end{aligned}$$

### Antifungal assay via radial growth

To determine the effect of the tested nanomaterials on fungi, *Aspergillus* was used. Dextrose agar culture media for fungi were prepared to perform the antifungal assay. Specific solid medium containing different amounts of the tested nanomaterials were established. Before being used in Petri dishes, the different treatments were mixed to ensure a good dispersion of the tested nanomaterials in the culture medium. We punched a 1 cm diameter hole in the middle of every solidified culture medium. In order to gain homogeneity and reproducibility in the experiment, the fungal spores were collect from the same 15 day old *Aspergillus* fungi parent. Subsequently, 1000 conidia in 50 μL PSB were added to the center hole of each Petri dish containing the culture medium for each of the described treatments. The dishes were move to the culture oven, which was held constant at 25 °C. From the fifth day, a photographic record was taken for each fungi sample. These records were analyzed with Image-J to measure the growth area, and we analyzed the growth rate of each treatment in comparison to the control sample. In addition, the synergy of the nanomaterials with the commercial antibiotics (amphotericin B and itraconazole) was studied. First of all, we tested the optimized concentration of pure antibiotics, and then 0.5 mg/L amphotericin B and 6.0 μg/mL itraconazole were used as the basis for the enhancement study.

2 $${\text{Growth Rate of ZnO Treatment}} (\% ) = \frac{{\text{Growth of ZnO Treatment}}}{{\text{Growth of Control}}} \times {\text{ 100}}\%$$

### ROS detection

A DCFDA Cellular ROS detection assay kit and a modified DCFH-DA (2′,7′-Dichlorofluorescin diacetate) method were used for measurement of reactive oxygen species in cellular and non-cellular settings, respectively [34]. (i) Cellular study: following the DCFDA kit’s manufacturer's instructions, L929 cells (10,000 cells per well) were cultured in 96-well micro-plates and incubated at 37 °C in a 5% CO_2_ atmosphere for 24 h to allow adherence. Cells were washed with 1 × buffer and then we added the tested materials (DE-ZnO). Meanwhile, we stained the cells with 100 μL DCFDA (25 μM, in 1 × kit dilution buffer) for 45 min at 37 °C in a humidified 5% CO_2_ incubator. Then, the DCFDA solution in the well was replaced with 1 × buffer. The basal ROS production was detected by recording the fluorescence (Ex/Em: 485/535 nm) with a micro-plate reader. (ii) Non-cellular study: 4 mg dichlorofluorescein (DCF) was added to 1 mL of phenol at 1/1000 DW dilution to obtain a concentration of 10 μM DCF. Then, 100 μL of DCF was added to the wells in dark, clear bottom 96-well microplates. Fluorescence readings were taken after 1 h incubation at 37 °C in a humidified 5% CO_2_. Then, we added the test materials (DE-ZnO) and fluorescence readings were taken every 30 min during incubation at 37 °C in a humidified 5% CO_2_ atmosphere. Relative fluorescence units (RFU) were calculated from all measurements. The plates were kept at 37 °C in a humidified 5% CO_2_ incubator in the dark between readings. Fluorescence was measured using the following settings: excitation (Ex) at 495 nm, emission (Em) at 530 nm; temperature 37 °C; reading mode: bottom) including the background as negative controls. Each experiment was repeated on three separate occasions (n = 3). Relative fluorescence units (RFU) were calculated from all measurements. The plates were kept at 37 °C in a humidified 5% CO_2_ incubator in the dark between readings.

### In vitro cytotoxicity assay

A colorimetric assay kit (Cell Counting Kit-8) was used to measure the cytotoxicity of our materials [11]. Firstly, L929 and HCT-116 cells were cultured in DMEM medium supplemented with 10% FBS at 37 °C in an atmosphere with 5% CO_2_ and 95% relative humidity. Cells were seeded on 96-well plates at a density of 5 × 10^4^ cells/well for 24 h to allow cell attachment. The medium was removed and the cells were washed once with PBS. The test materials (optimized ZnO modification ratio test) at certain concentrations in DMEM were added to separate wells in quadruplicate and incubated with the cells for another 24 h. Corresponding samples of DE-ZnO at concentrations of 0.5, 1.0, 1.5, 2.0, 2.5 and 3.0 mg in DMEM were subjected to the same process. After 24 h incubation, the suspensions were removed and the wells were washed once with PBS. A 100 μL sample of WST-8 (0.5 mg/mL in culture medium) was then added, being careful to not introduce bubbles into the wells, since they interfere with the O.D. reading. Then, the plates were incubated for 4 h at 37 °C in 5% CO_2_ prior to analysis. We measured the absorbance at 450 nm using a micro-plate reader (BioTek, US). Absorbance values for the untreated cells were taken as controls (100% survival). Cell viability was then calculated according to the following equation:

3 $${\text{Cell Viability (\% ) }} = \frac{{\text{OD}_\text{treatment}}}{{\text{OD}_\text{No Treatment}}} \times 100\%$$

The biocompatibility of the ZnO nanomaterials and DE were confirmed through cell viability using an MTT assay.

### In vivo toxicity assay

ICR mice (6 weeks old, 15 males and 15 females) were purchased from Koatech (Gyeonggi-do, Korea). The mice were maintained at room temperature (22 ± 2 °C) with a 12/12 h light/dark cycle and were fed ad libitum. Thirty mice were randomly divided into three groups as follows: group I (control group); group II (LD-DE-ZnO), which received the LD-DE-ZnO; and group III (HD-DE-ZnO), which received the HD-DE-ZnO. Each group (n = 10) contained an equal number of male and females. Mice of each sex were administered 300 μL of DE-ZnO solution at dosages of 2 mg/ml (low dosage), 20 mg/mL (high dosage), or Milli-Q water (control group) by oral gavage. After administration, the animals were weighed and observed daily to detect any signs of toxicity over 14 days. After 14 days, the animals were euthanized using Zoletil (Virbac Korea, South Korea) and blood samples were collected from the aorta ventralis. The biochemical measurements consisted of analyses of the following: alanine aminotransferase (ALT), aspartate aminotransferase (AST), total bilirubin (T-Bil), blood urea nitrogen (BUN), creatinine, total protein (TP), albumin, albumin/globulin (A/G) ratio, total cholesterol (TC), triglyceride (TG), glucose, and phosphorous. Biochemical analysis of plasma samples was carried out by using commercially available kits according to the manufacturer’s instructions. After collection of the blood samples, the body surface, all orifices, the head, all internal organs of the abdominal and thoracic cavities, and their contents were visually observed for any signs of gross abnormalities. Body and organ weights were measured for each mouse. For grouped organs, the sum of those organs was used. After collection of the organs, including the brain, heart, lung, liver, kidney, spleen, testicle, or ovary, they were preserved in 10% phosphate-buffered formalin solution for histopathologic examination. Liver sections of 3 μm thickness were stained with hematoxylin and eosin (H&E). The slides were observed under an optical microscope **(**BX51, Olympus, Tokyo Japan). The experimental protocol and this study were approved by the Institutional Animal Care and Use Committee of the Laboratory Animal Center, Osong Medical Innovation Foundation (KBIO-IACUC-2020–011).

## Results

### Synthesis and characterization of DE-ZnO

**Fig. 1:**
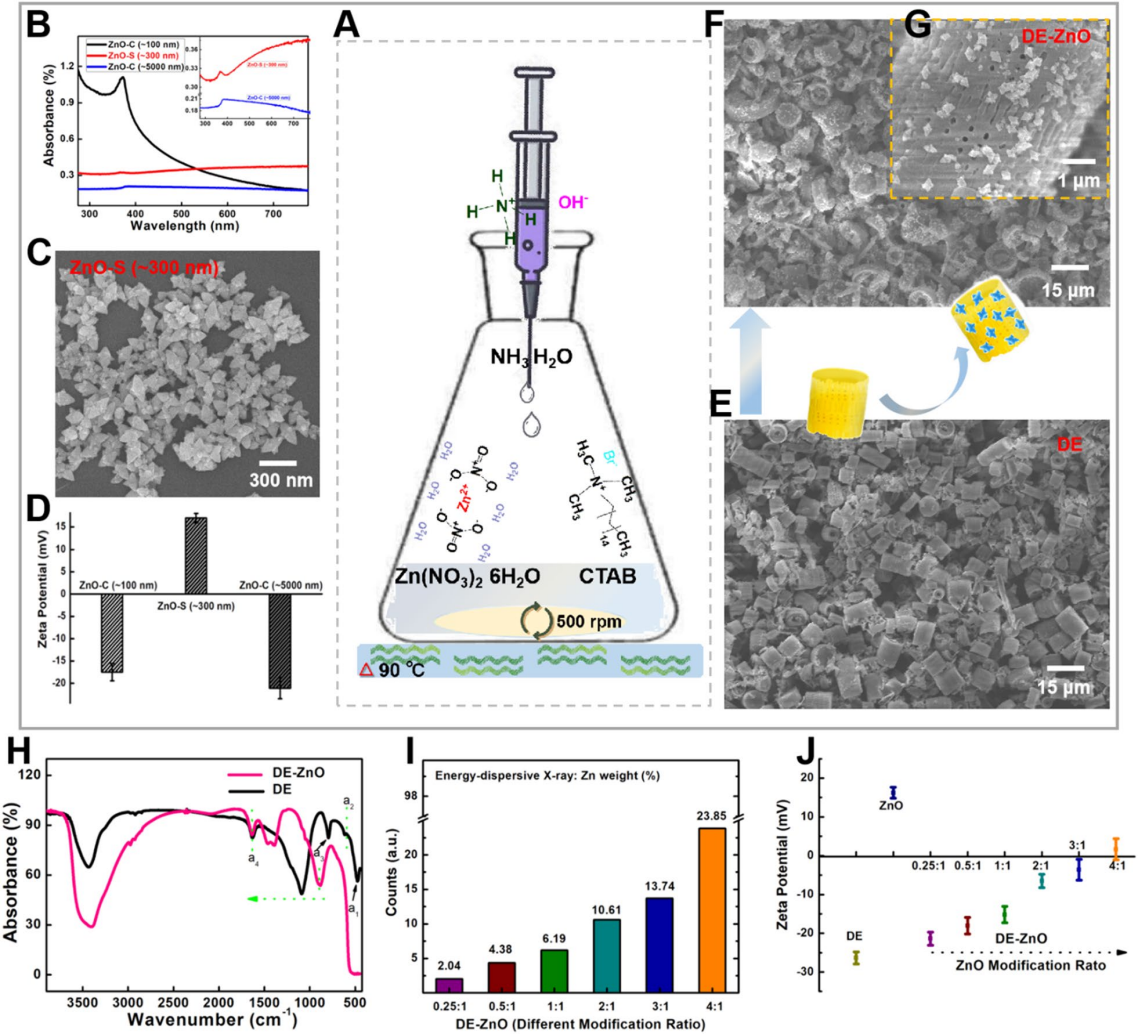
Study the mild-simple synthesis procedure and chemical characterization of nano-composite (DE-ZnO). **A** Illustration of the mild-simple synthesis procedure of DE-ZnO in middle of the graphic and surrounding with the property characterization. **B** UV–visible absorption spectra for ZnO nanomaterials (ZnO-S, commercially available ZnO nanomaterials; ZnO-C (~ 100 nm) and ZnO-C (~ 5000 nm)). **C** SEM images of ZnO-S (~ 300 nm). **D** Zeta potential of the ZnO nanomaterials. **E** SEM image of the precursor diatomaceous earth (DE). **F** SEM images of the product semiconductor nano composites DE-ZnO, inside **G.** is the amplification SEM image of the DE-ZnO surface. **H** FTIR spectra of DE-ZnO nanocomposites and pure DE. **I** Energy-dispersive X-ray of DE-ZnO at different modification ratios of ZnO:DE = 0.25:1; 0.5:1; 1:1; 2:1; 3:1; and 4:1, the percentage of ZnO are 2.04%, 4.38%, 6.19%, 10.61%, 13.74% and 23.85%, respectively. **J** Zeta potentials of DE-ZnO composites depend on the different ratios of ZnO on DE (ZnO:DE = 0.25:1, 0.5:1, 1:1, 2:1, 3:1, and 4:1). Error bars indicate the standard error of the mean based on at least three independent experiments

First, in order to synthesize ZnO-S (~ 300 nm) through facile hydro-thermal method (Fig. 1A), we optimized the one-pot method which reduce the cost of synthesis production using the zinc contains with 98% SiO_2_. The properties of our synthesized ZnO-S (~ 300 nm) were referred to commercial ZnO nanomaterial [named ZnO-C (~ 100 nm) and ZnO-C (~ 5000 nm)] [35]. Through UV–visible absorption analysis (Fig. 1B), the characteristic peak at 381 nm confirmed the ZnO and the half-peak angles of the black (ZnO-C ~ 100 nm) and red (ZnO-S ~ 300 nm) spectrum show the uniform structure form. The morphology of these nanomaterials (ZnO-S in Fig. 1C and ZnO-Cs in Additional file 1: Fig S1A-B) was confirmed by scanning electron microscopy (SEM). Then, the surface performance of these nanomaterials was confirmed by Zeta potential (Fig. 1D) and Fourier-transform infrared spectroscopy (FTIR) (Additional file 1: Fig S1C). The special positive surface charge of the synthesized ZnO-S occurred by coating it with NH_4+_ groups. In Additional file 1: Fig S1C, the particular peaks of the red spectrum at a_1_(735 cm^−1^), a_2_(1009 cm^−1^), and a_3_(1075 cm^−1^) for ZnO-S showed the N–O bands, which may come from NH_4_OH synthesis process as the previous report [36]. The positive surface charge of the ZnO-S (~ 300 nm) provided an optimum condition for further biocompatibility applications. Due to the surface charge of the most bacteria and fungi supposed to be negative [4, 37]. Thereby, the ZnO-S with the positive surface charge would attract the bacteria and fungi to be close and it is good for the materials to play antibacterial and antifungal effect. Through Barrett-Joyner-Halenda (BJH) pore size and volume analysis (Additional file 1: Fig. S1D–F), the mild manufacturing process used for ZnO-S synthesis was optimized to obtain uniform ZnO-S, and the morphology of the ZnO-S was built by nanoparticles (3 ~ 7 nm), which implied that there is a large surface area. Its large surface area and the surface charge of the synthesized ZnO-S were considered to be beneficial. Using the synthesized ZnO-S, we compared its antibiotic effect against bacteria and fungi with different amounts of commercialized ZnO nanomaterials (6 ~ 14 μg/mL). For the antibacterial effect tests (Additional file 1: Fig. S1G), the nanomaterials were mixed with *E. coli* in lysogeny broth (LB). After incubation for 12 h, the bacterial survival rate was calculated. The ZnO-S was a good candidate with a stronger antibacterial effect as compared to those of the commercialized ZnOs (Additional file 1: Fig. S1G). For the antifungal effect testing, the nanomaterials (10 µg/mL) were mixed with *A. fumigatus* in culture plates. Then, we analyzed the diameter of the grown microflora after 7 days of culture (Additional file 1: Fig. S1H, I and S2). The antifungal activity of ZnO-S is better than that of commercial ZnOs due to the shape of ZnO-S, which can efficiently break the cell membrane of fungi. Based on these results, the antibiotic activity of ZnO-S is promising, but there is still not enough persistence and efficacy for it to be a good candidate for antimicrobial treatment.

Finally, to maximize the efficacy and persistence of its antibiotic activity, we designed a novel non-toxic composite (called DE-ZnO) combined with ZnO-S and frustules diatomaceous earth (DE, Fig. 1E), which has a naturally assembled amorphous silica architecture with countless nanosize pores and abundant active hydroxyl groups on its substantial surface. A DE framework semiconductor composite (DE-ZnO) is obtained due to the precursor Zn^2+^ settling on the DE due to the surfactant (CTAB) using the facile hydro-thermal method (Fig. 1F). The ZnO-S is well distributed across the surface of DE (Fig. 1G). Hence, several in vitro and in vivo tests were conducted to verify the performance of the DE-ZnO composites. To confirm the properties of the bio-inspired DE-ZnO composites, the FTIR of the DE and DE-ZnO composites was tested (Fig. 1H). The missed peaks of DE-ZnO at a_1_ 495 cm^−1^, a_2_ 650 cm^−1^, and a_3_ 750 cm^−1^ as compared with DE were caused by modification with the NH_4_OH reaction. On the other hand, the interaction between the grown ZnO and DE framework were confirmed with the red shift. The decreased wavenumber shows that the DE-ZnO (1152 cm^−1^) composites are much more active than pure DE (1152 cm^−1^). Next, to optimize the reaction ratio between the ZnO and DE, the parallelism reaction ratios of ZnO:DE = 0.25:1; 0.5:1; 1:1; 2:1; 3:1; and 4:1 were examined. The energy-dispersive X-ray of the DE-ZnO showed that the percentages of ZnO on the DE are 2.04%, 4.38%, 6.19%, 10.61%, 13.74%, and 23.85%, corresponding to each parallelism reaction ratio (0.25, 0.5, 1, 2, 3, and 4), respectively (Fig. 1I and Additional file 1: Fig. S3). Meanwhile, the parallelism zeta potential of the DE-ZnO composites confirmed that the surface charge of the DE-ZnO was positive and correlated with the amount of coated ZnO-S (Fig. 1J). Due to the well-washed DE having a negative surface charge and the synthesized ZnO-S having a positive surface charge, the DE-ZnO composites can be changed to a positive charge by adding a sufficient amount of ZnO-S. Thus, the integral surface charge of the DE-ZnO composites could be neutralized between the substrate DE (-) and the growing ZnO-S (+), which were mainly owing to the natural stable property of the DE and the semiconductor properties of ZnO-S. Based on these results, the DE-ZnO composites were well-conjugated.

### Antibacterial study of DE-ZnO

**Fig. 2:**
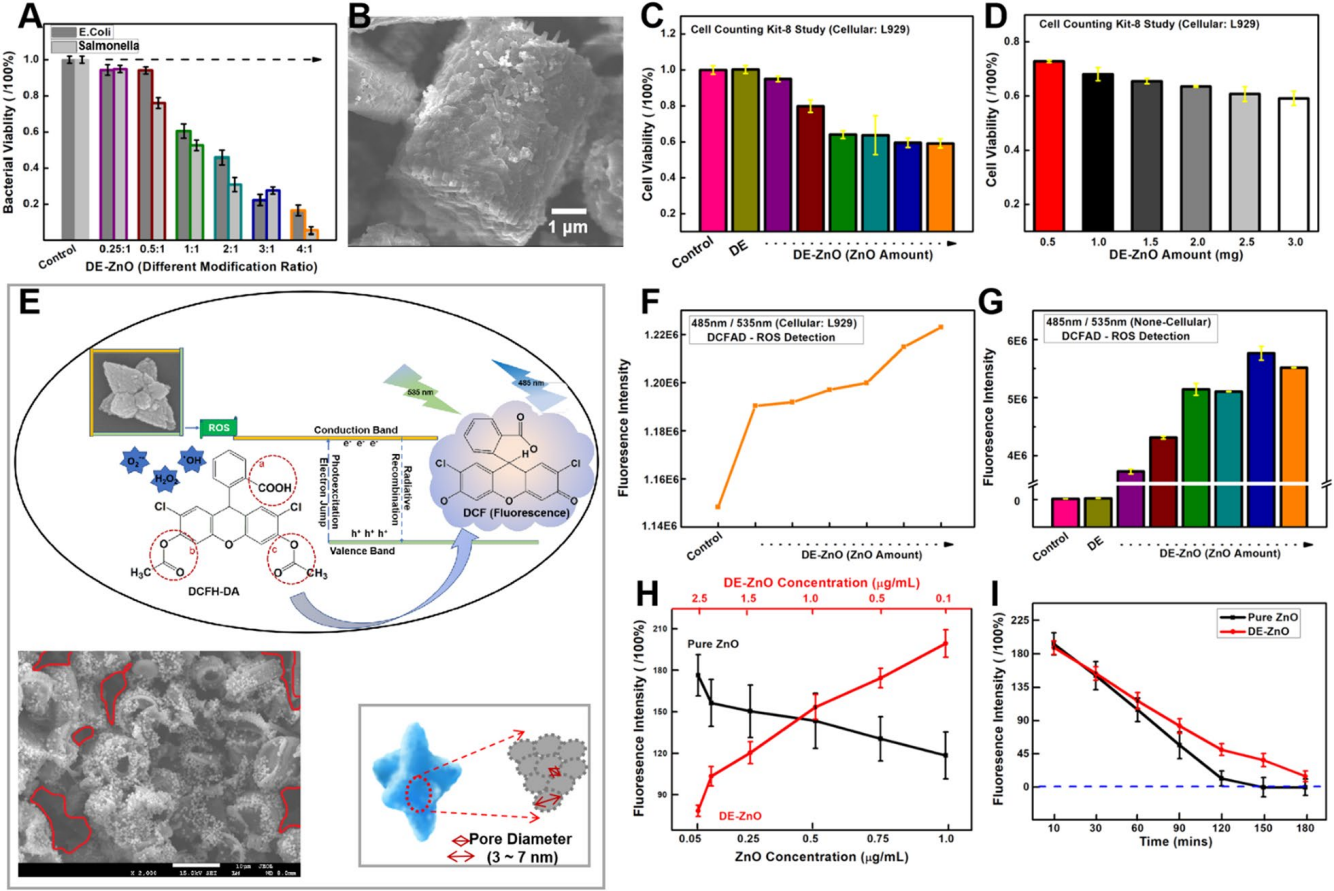
Study the antibacterial activity and bio-safety dosage of DE-ZnO. **A** Antibacterial efficiency of DE-No against Gram-negative bacteria (*E. coli, S. enterica*) at a concentration of 10 µg/mL. **B** SEM image showing the surface of DE-ZnO composites after meeting/absorbing pathogen. **C** Cell viability study of the cytotoxicity of DE-ZnO at different modification ratios with dosage of 0.25 mg in one hole through Cell counting Kit 8 method. **D** Dosage depended cytotoxicity study of DE-ZnO (ZnO: DE = 2:1, 10.61% of ZnO), L929 cells were exposed to different doses (0.5–3.0 mg) for 24 h. Each data value is mean ± SE of three independent experiments. **E** Schematic illustration of the Reactive oxygen species (ROS) detection, due to the band gap of semiconductor could absorb energy and release ROS which would transfer the DCFH-DA to DCF (Fluorescence); inside the SEM image of DE-ZnO confirmed that the well-wash DE-ZnO composites is without dispersed ZnO-S (blank in red cycle); meanwhile the special multi-pore morphology of ZnO-S has been marked. **F** Cellular ROS detection assay using DE-ZnO with different ratios of ZnO modification (ZnO:DE = 0.25:1, 0.5:1, 1:1, 2:1, 3:1, and 4:1). **G** Non-cellular ROS detection assay using DE-ZnO with different ratios of ZnO modification (ZnO:DE = 0.25:1, 0.5:1, 1:1, 2:1, 3:1, and 4:1). **H**, **I** The mechanisms of the delay ions/ROS ‘quenching’ related antifungal property of DE-ZnO. **H** Non-cellular ROS detection assay using ZnO and DE-ZnO with different concentrations of nanomaterials, showing the relatedness between the pure ZnO and DE-ZnO. **I** Time-depended study of the effect from ROS protection, explaining the ‘quenching’. DCF, 2′,7′-Dichlorofluorescein; DCFH-DA, 2′,7′-Dichlorofluorescin diacetate, Error bars indicate the standard error of the mean based on at least three independent experiments)

Using the DE-ZnO composites, we tested their antibacterial effect against Gram-negative bacteria (Fig. 2A). For these tests, a suspension of Gram-negative bacteria (*E. coli* and *S. enterica*) was used to evaluate the antibacterial activity of 10 µg/mL DE-ZnO into parallelism reaction ratio. The DE-ZnO composites were added into a medium tube containing 1 × 10^7^ CFU/mL bacteria (*E. coli* or *S. enterica*) suspension. After incubation for 16 h at 37 °C shaking at 210 rpm, the optical density of each sample at OD 600 nm was measured to check for bacterial viability. The results showing that the antibiotic effect of DE-ZnO composites were enhanced as the ratio of ZnO increased, the higher ratio of the DE-ZnO composites showed higher antibacterial properties than the others. In Fig. 2B, the DE-ZnO composites absorbed the bacteria entirely which could facilitate to break the cell membrane in a minimum area.

Due to the high toxicity of commercial drugs, the effective treatment of patients with fungal and bacterial infections has been extremely limited. Thus, we investigated the cytotoxicity of DE-ZnO at different modification ratios through Cell counting Kit 8 method with the non-cancerous L929 cell (Fig. 2C). The cell viability is relatively stable (70 ~ 60%) at the 10.61% (2:1) ratio of DE-ZnO and is little affected at amounts up to 3 mg (Fig. 2D), which is the higher dosage of the DE-ZnO composite for in vitro study (Table 1) [2, 32]. According the EDX study, we estimated the amount of ZnO in DE-ZnO composites is about 10% of weight of DE-ZnO. 300 μg/mL of ZnO in DE-ZnO composites is 30 times higher than the pure ZnO, showing lower cytotoxicity of DE-ZnO. In order to further explore the efficiency and biocompatibility of DE-ZnO, we performed a DCFDA-DA Cellular ROS detection assay for measurement of ROS in cellular and non-cellular environments containing the DE-ZnO composites. A simple schematic diagram showing in Fig. 2E for cellular environment testing, esterases cleave DCFH-DA at two ester bonds, which could produce a relatively polar and cell membrane-impermeable product (H_2_DCF), this non-fluorescent molecule accumulates in cells and subsequent oxidation yields the highly fluorescent product DCF. In non-cellular environments, when reverse detection was applied, a fluorescent DCF solution was prepared and its fluorescence intensity was protected in the presence of ROS. The redox state of the sample can be monitored by detecting the intensity of the fluorescence in the presence of ZnO-S. We observed that the ROS from DE-ZnO composites can be induced when it interacts with cells (Fig. 2F). In the non-cellular study, the pure DE did not affect the fluorescence of DCF, but the presence of DE-ZnO composites did slow down the speed of fluorescence quenching (Fig. 2G). Then, the DE-ZnO composites with different ratios of ZnO modification (ZnO:DE = 0.25:1, 0.5:1, 1:1, 2:1, 3:1, and 4:1) were compared, and the ZnO amount-effect on ROS release was verified (Fig. 2G). We performed a non-cellular DCF fluorescence study with the DE-ZnO (0.1 to 2.5 μg/mL) composites (Fig. 2E) and the pure ZnO-S (0.05 ~ 1.0 μg/mL) to check the amount-effect for 30 min. As the fluorescence effect plots show, the fluorescence intensity from 0.5 μg/mL ZnO-S was similar to that of 1.0 μg/mL DE-ZnO (Fig. 2H). Then, certain amounts of pure ZnO-S (0.5 μg/mL) and DE-ZnO (1.0 μg/mL) were applied to the time linear record test of the DCF fluorescence. Obvious fluorescence quenching was detected and the fluorescence quenching of the DE-ZnO composites was slower than that of the pure ZnO (Fig. 2I). This phenomenon indicated that the ions and ROS released from the DE-ZnO composites have been through an ‘absorb-release’ course from the porous framework, which has prolonged the active process.

**Table 1:** Comparison of the cytotoxicity of the DE-ZnO with other ZnOs

Safety dosage	Cytotoxicity (IC_50_)	Oral (Rat)
ZnO	~ 10 µg/mL [39]	5 mg/kg
SiO_2_	500 µg/mL [40]	–
SiO_2_-ZnO	100 μg/mL [41]	–
DE-ZnO (Our work)	3.0 mg/mL	300 mg/kg

### Antifungal study of DE-ZnO

**Fig. 3:**
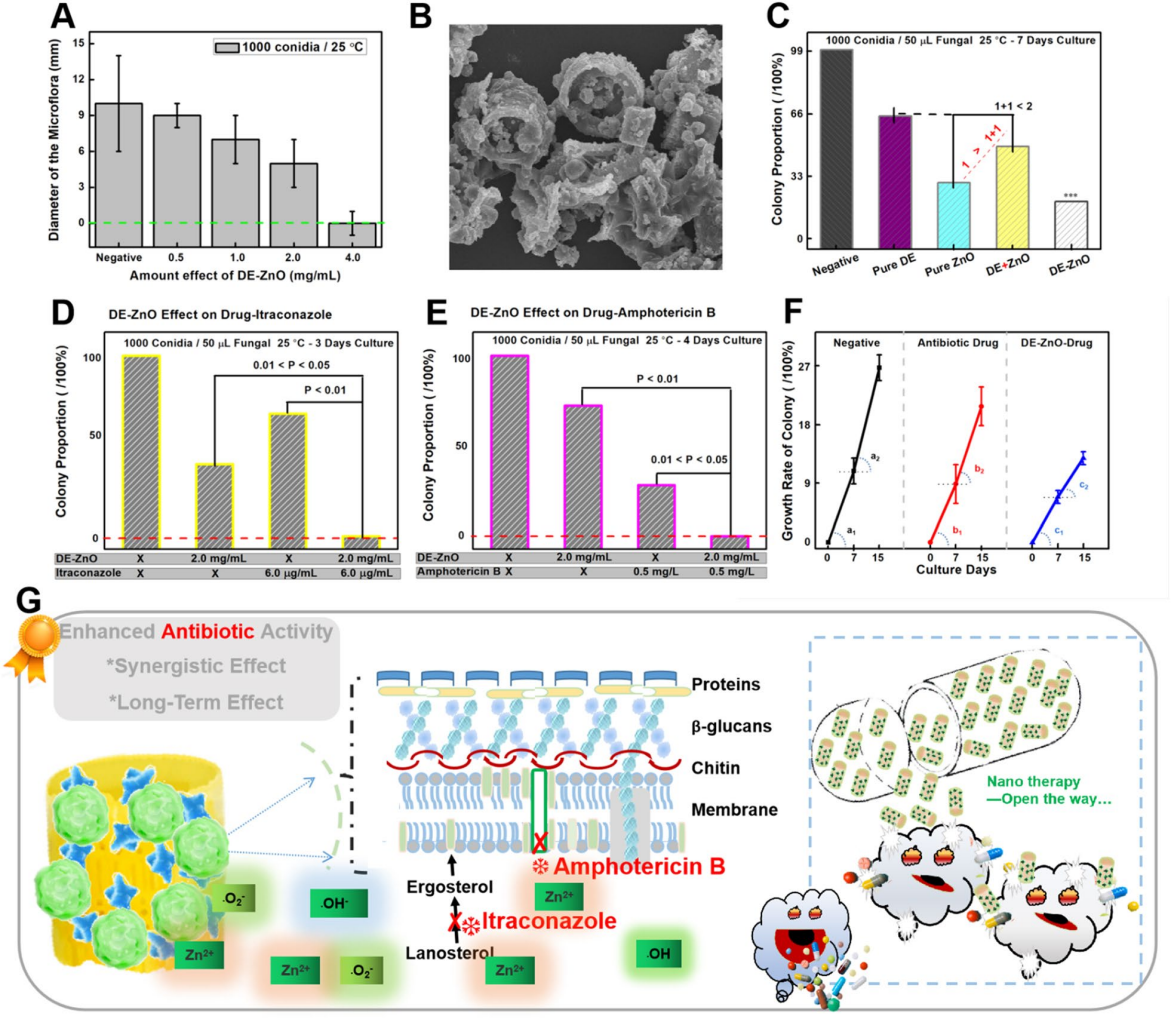
Study the synergistic effect and persistence of DE-ZnO composites against fungal infection. **A** Amount effect of DE-ZnO on anti-fungal activity (1000 conidia, 25 °C, culture for 3 days). Each data value is mean ± SE of duplicate independent experiments with 3 days culture. **B** SEM image of fungal aspergillus absorbed on the DE-ZnO composites. **C** Growth tendency of fungi with a synergy effect between DE + ZnO and pure ZnO showing a “1 + 1 < 1” preference. Each data value is mean ± SE of duplicate independent experiments. **D** Antifungal activity of the DE-ZnO composite, itraconazole, and combination groups up to 3 days of culture with 1000 conidia. **E** Antifungal activity of the DE-ZnO composite, amphotericin B, and combination groups up to 4 days of culture with 1000 spores. Each data value is mean ± SE of duplicate independent experiments. **F** Long term (15 days) culture of the fungi; growth rate of the colony reflects the enhanced synergy effect of the DE-ZnO composites; growth tendency of the synergy effect between the DE-ZnO composites and commercial drugs (*Amphotericin B and Itraconazole*). **G** Scheme of synergistic effect from a combination of DE-ZnO and the existing drugs as a nano therapy way

Next, the treatment problem of the fungal infection inspired us to study the antifungal efficacy with the DE-ZnO semiconductor composites. As we previously reported, ZnO induces the generation of reactive oxygen species (ROS: O^2•‑^, ·OH, H_2_O_2_) that react with the peptidoglycan layer and break the glycosidic bonds in a biological manner. Here, we tested the DE-ZnO (2:1, 10.61% of ZnO) with different concentration ranges from 0.5 to 4.0 mg/mL on *A. fumigatus* plates (1000 conidia, 25 °C). We monitored the fungal growth condition within 14 days, the diameter of the grown microflora at the 7th day was measured (Fig. 3A and Additional file 1: Fig. S4). Under the culture condition, the fungi were barely growing at 4.0 mg/mL of the DE-ZnO composites. Thus, the DE-ZnO composites were not only inhibiting the fungal growing, but also kill the fungal activity due to break the cell wall of fungal.

Furthermore, to investigate the effect of DE-ZnO on antifungal, we added the DE-ZnO composite into the fungal spore solution for 3 min. The DE-ZnO is well absorbed the fungal spores (Fig. 3B) due to the capture enrichment property of DE-ZnO composite, which is an important advantage for its antifungal property. For more insights into this highly cooperative relationship and synergistic effect of the DE-ZnO, we treated fungi cultures with pure DE, pure ZnO, ‘pure DE + pure ZnO’ and DE-ZnO composites (Fig. 3C). The radial growth estimations indicate that (i) The colony growth when treated with pure ZnO and DE-ZnO composites are well matched with the fluorescence quenching study, with a significant synergy effect of the DE-ZnO composites showing “1 + 1 > 2” performance; and (ii) The antifungal effect of ‘pure DE + pure ZnO’ is less than the DE-ZnO composites, even less than the same amount of pure ZnO (“1 + 1 < 1”), and this phenomenon is consistent with a prior report of N dopants into hierarchically porous composites (Fig. 3C) [31, 38].

### Synergistic effect and persistence of non-toxic DE-ZnO composites

Nowadays, it has become harder for antibiotics to break cell membranes, due to the development of resistance by the cell wall that keeps antibiotics out of fungi and Gram-negative bacteria. Thus studies designed to combat the resistance of fungal, we tested the synergistic effect and persistence of DE-ZnO composites in combination with commercial antibiotics (amphotericin B and itraconazole) for their antifungal activity. For a more detailed investigation into the synergistic effect between the DE-ZnO composites and the commercial drugs, 2.0 mg/mL DE-ZnO with either 0.5 mg/L amphotericin B or 6.0 μg/mL itraconazole were used as the initial concentrations. We showed significant enhancements in both (1) the DE-ZnO with itraconazole (Fig. 3D) and (2) the DE-ZnO with amphotericin B groups (Fig. 3E). The mechanisms of the antifungal activity by the antibiotics have been previously demonstrated Itraconazole disrupts the conversion of lanosterol to ergosterol, which disrupts the growth process, and amphotericin B actively penetrates the cell membrane.

To gain insight into the antibiotic persistence of the DE-ZnO composites, long term culture of the fungi was investigated. The growth of colonies from the negative control, antibiotic drug treatment only, and DE-ZnO with antibiotic drug groups was evaluated and the growth rates on days 7 (a_1_ ~ c_1_) and 15 (a_2_ ~ c_2_) have been recorded (Fig. 3F). According to the slopes of the groups for the growth rate, “a_1_ > b_1_ > c_1_” demonstrated that the DE-ZnO composites enhanced the antifungal performance of those antibiotics. “a_2_ > a_1_; b_2_ > b_1_” indicated that the fungi were growing faster after 7 days of culture in both the negative and antibiotic treatment only groups. However, the DE-ZnO composites combined with the antibiotic exhibited a striking long-enduring inhibition, with “c_2_ < c_1_”, which suggests a strong synergistic effect.

Thereby, the antibiotic efficacy and synergy effect of DE-ZnO composites with the commercial drugs (Fig. 3G) shown that the long-term effect as an antifungal is due to the delayed release of ions and ROS reactions. First, the capture-enrich step between the cell wall of fungal and the activated surface of DE-ZnO composite can be achieved through the surface charge adsorption and van der Waals forces. Second, the nano-lysis step of the DE-ZnO can be achieved to the fungi by breaking the cell membrane. Taken together, the DE-ZnO composites are promising and are good candidates for antifungal and antibacterial treatment. Therefore, we concluded that the synergistic effect and persistence of DE-ZnO composites could be used as a promising antifungal agent to solve the inconvenience caused by the requirement for multiple doses of insoluble antibiotics. To a certain extent, the nano-therapy open a way for antibiotic treatment to be reduced the usage of antibiotic and decelerated the fungal mutations.

### The biocompatibility and toxicity of DE-ZnO composites in vivo

**Fig. 4:**
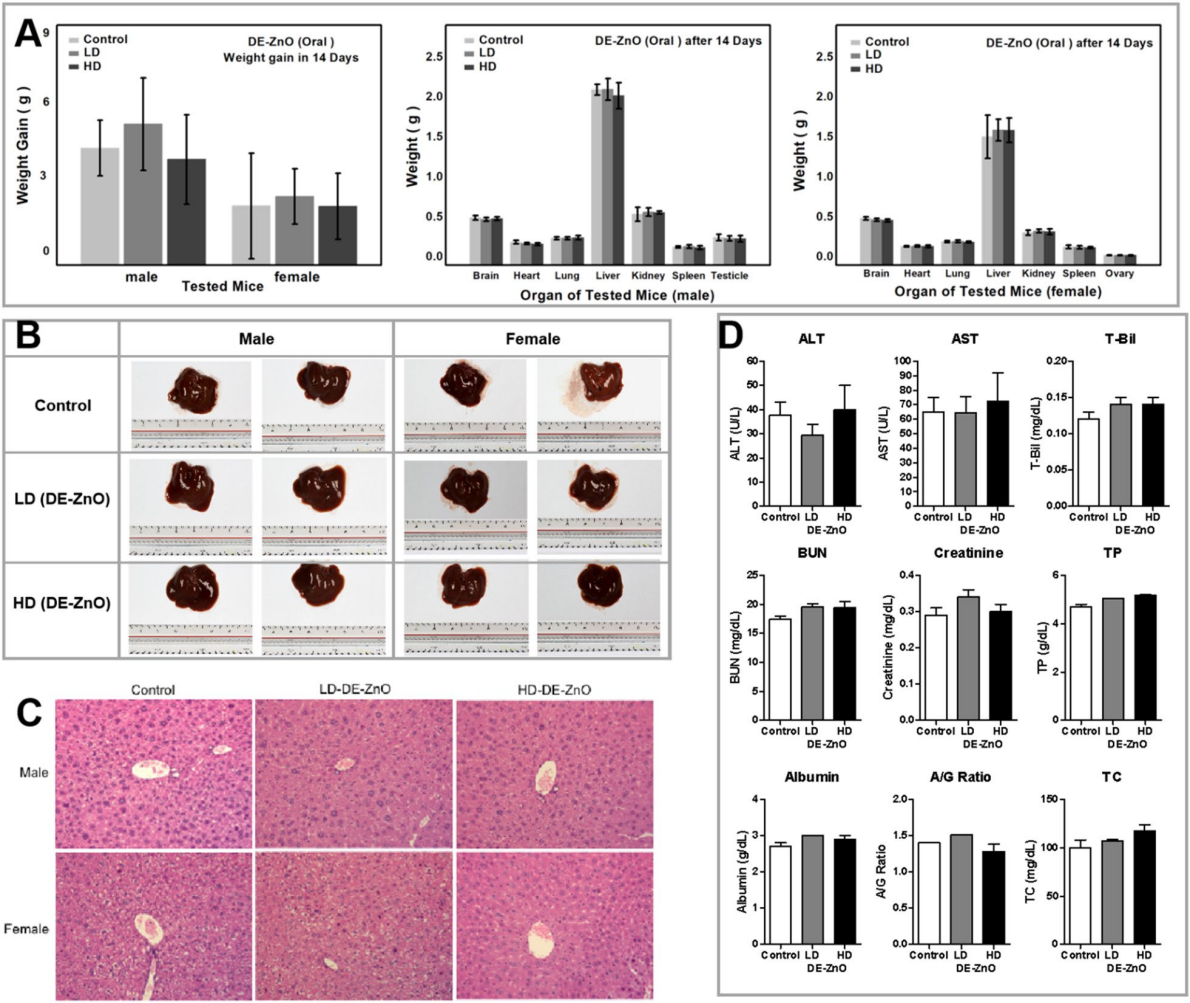
Study the biocompatibility and toxicity of DE-ZnO composites in vivo. **A** Body weight and major organs weight changes of the mice treated with LD-DE-ZnO or HD-DE-ZnO composites by oral administration, monitored for 14 days. male and female mice after 14 days of oral administration with DE-ZnO composites. **B** Liver morphology changes in the mice after 14 days of oral administration with DE-ZnO. **C** Biochemical parameters changes in mice treated with LD-DE-ZnO or HD-DE-ZnO composites by oral administration. **C** Histology analysis of liver tissue of mice treated with LD-DE-ZnO or HD-DE-ZnO composites by oral administration. **D** The sections were stained with hematoxylin and eosin (Magnification ×200). Histologic changes showed no differences among groups (n = 4 mice per group). Each data value is mean ± SE. (n = 4 mice per group). ALT: alanine aminotransferase; AST: aspartate aminotransferase; T-Bil: total bilirubin; BUN: blood urea nitrogen; TP: total protein; A/G: albumin/globulin; TC: total cholesterol; LD: low dosage; HD: high dosage

We next evaluated the in vivo toxicity of the DE-ZnO composites. After DE-ZnO administration, all animals were observed daily for clinical signs of toxicity, including tremors, convulsions, salivation, nausea, vomiting, diarrhea and body weight changes, and death, during the 14 days (Fig. 4). There were no deaths at both dosages. There were no significant alterations of weight or any toxicity symptoms in both the low dosage (LD)-DE-ZnO and high dosage (HD)-DE-ZnO groups. Meanwhile, when we compared with same dosage of the nanomaterials, the toxicity of DE-ZnO (2:1) is 6 times lower using oral medication and the dosage amount of DE-ZnO is 3 times higher than the SiO_2_-ZnO (Table 1) [34, 38]. And the animals were sacrificed after 14 days, and their major organs including brain, heart, lung, liver, kidneys, spleen, testis or uterus were collected and weighed for males and females (Fig. 4A and Additional file 1: Fig. S5). There were no differences among the DE-ZnO and control groups. Meanwhile, examination of the organs showed that there were no abnormalities in any of the tested groups. Moreover, we did not observe any histopathological changes or damage in the liver of DE-ZnO treated mice (Fig. 4B, C). Furthermore, the results of the biochemical analysis conducted 14 days after the administration of the DE-ZnO are shown in Fig. 4D and Additional file 1: Table S1. There were no differences in the biochemical parameters of the DE-ZnO groups compared to the control group. These results revealed that there were no signs of toxicity in the mice treated with low or high doses of DE-ZnO by oral administration in vivo. These findings suggest the DE-ZnO composites are relatively safe for biomedical applications.

## Discussion

To the best of our knowledge, we are the first to synthesize a novel composite conjugated ZnO on the porous surface of DE for an optimal antibiotic effect with non-toxicity (Table 1). Based on our study of its morphological changes, we observed that the DE-ZnO composites strongly bound to the pathogen’s membrane and disrupted its membrane morphology. Generally, disruption of the fungal cell wall is more difficult than for other pathogens, which subsequently leads to low antibiotic effects. Using multiple experiments, we demonstrated several possible mechanisms whereby DE-ZnO enhances antibiotic activity and persistence against fungi (*A. fumigatus*) and Gram-negative bacteria (*E. coli* and *S. enterica*): (1) Increasing physical attraction. The surface charge of the composites is positive due to the combination of two physical functionalities by the sharp shape of the synthesized ZnO and the porous structured DE for enhancement of physical disruption and physical absorption, respectively. (2) Increased ROS production and long persistence. The DE-ZnO composites lead to increased ROS production, and the effect can enhance antibiotic activity at the cellular and non-cellular level for longer periods than other ZnO materials. (3) Excessive zinc ions (Zn^2+^). Zn^2+^ can directly rupture the cell wall when the composites bind to pathogens. Additionally, we evaluated the toxicity of DE-ZnO after oral administration to mice. While further studies are desirable to fully establish the safety of DE-ZnO in mice and humans, we observed the composite was safe at experimental dosages when administered to mice for 2 weeks. Their organs had normal weights and their blood chemistry was normal as compared to the controls.

Next, we systemically investigated the synergistic effect between the DE-ZnO composites and existing antifungal agents (amphotericin B and itraconazole) using *A. fumigatus.* These antifungal agents have many limitations such as high toxicity, insolubility, sensitivity to pH, and severe side effects for patients with cancers and transplants. The antifungal efficacy of DE-ZnO combined with either of these antifungal agents was significantly enhanced compared to that of the single treatment. Even though the existing antifungal agents are toxic to the organs in the mouse, a reduced dose of the existing antifungal agent made possible by the addition of DE-ZnO can reduce their toxicity and enhance their antifungal activity. Although further study is required to verify the ability of DE-ZnO to kill pathogens in infected mice and humans over the long-term, this synergistic therapy describing the potential of DE-ZnO to enhance antifungal activity alone or in combination therapy with lower doses of existing drugs has great clinical potential.

Our results have shown that this non-toxic DE-ZnO composite can be used as an antibiotic agent against fungi and Gram-negative bacteria in a clinical setting. Moreover, the DE-ZnO composites have a porous structure, which can be efficiently loaded with another antibiotic agent, so they can also be used as carriers for drug delivery. Hence, given recent advances in the surface chemistry of nanomaterials, future study will focus on drug delivery systems containing DE-ZnO composites to develop efficient therapies against target sites in various clinical applications.

## Conclusion

In this study, we synthesized the facile natural semiconductor composites (DE-ZnO) and demonstrated the antibiotic efficacy and persistence of DE-ZnO against fungi and Gram-negative bacteria. We found that the DE-ZnO composite had enhanced antibiotic activity against fungi (*A. fumigatus*) and Gram-negative bacteria (*E. coli* and *S. enterica*). Therefore, we envision that DE-ZnO composites can be used to enhance the mode of action of antibiotics against fungi and bacteria, and thus will be useful as novel antibiotic agents for fighting antimicrobial infections.

## Supplementary Information

**Additional file 1.** Additional Figures and Table.

## Data Availability

Not applicable.

## Abbreviations

DE: Diatomaceous earth
ZnO: Zinc oxide
ZnO-C: Commercial ZnO nanomaterial
ZnO-S: Synthesized ZnO nanomaterial
DE-ZnO: Composites
DCF: 2′,7′-Dichlorofluorescein
CTAB: Hexadecyltrimethylammonium bromide
DMEM: Dulbecco’s Modified Eagle’s Medium
LB: Luria–Bertani broth
FBS: Bovine serum
ZnNO_3_·6H_2_O: Zinc nitrate hexahydrate
NH_3_: Ammonium hydroxide solution
FE-SEM: Field emission scanning electron microscopy
DLS: Dynamic light scattering
FTIR: Fourier-transform infrared spectroscopy
CFU: Counting colony forming units
OD: Optical density
ROS: Reactive oxygen species
LD: Low dosage
HD: High dosage
ALT: Alanine aminotransferase
AST: Aspartate aminotransferase
T-Bil: Total bilirubin
BUN: Blood urea nitrogen
TP: Total protein
A/G: Albumin/globulin ratio
TC: Total cholesterol
TG: Triglycerides
HCT-116: Colorectal cancer cells
*E. coli*: *Escherichia coli*
*S. enterica*: *Salmonella enterica*
ICU: Intensive care unit
IAI: Invasive Aspergillosis infection
MRSA: Methicillin-resistant *S. aures*
COVID-19: Coronavirus disease 2019

## References

[1] Koehler P, Bassetti M, Chakrabarti A, Chen SC, Colombo AL, Hoenigl M, Klimko N, Lass-Flörl C, Oladele RO, Vinh DC (2020). Defining and managing COVID-19-associated pulmonary aspergillosis: the 2020 ECMM/ISHAM consensus criteria for research and clinical guidance. Lancet. Infect. Dis.

[2] Liu H, Zou Q, Qiao Z, Jang YO, Koo B, Kim MG, Lee HJ, Kim S-H, Shin Y (2021). facile homobifunctional imidoester modification of advanced nanomaterials for enhanced antibiotic synergistic effect. ACS Appl. Mater. Interfaces.

[3] Cornely OA, Alastruey-Izquierdo A, Arenz D, Chen SC, Dannaoui E, Hochhegger B, Hoenigl M, Jensen HE, Lagrou K, Lewis RE (2019). Global guideline for the diagnosis and management of mucormycosis: an initiative of the European Confederation of Medical Mycology in cooperation with the Mycoses Study Group Education and Research Consortium. Lancet. Infect. Dis.

[4] Edson JA, Kwon YJ (2016). Design, challenge, and promise of stimuli-responsive nanoantibiotics. Nano Convergence.

[5] Steenwyk JL, Mead ME, Castro PA, Valero C, Damasio A, Santos RA, Labella AL, Li Y, Knowles SL, Raja HA (2020). Genomic and phenotypic analysis of COVID-19-associated pulmonary aspergillosis isolates of *Aspergillus fumigatus*. BioRxiv.

[6] Borman AM, Palmer MD, Fraser M, Patterson Z, Mann C, Oliver D, Linton CJ, Gough M, Brown P, Dzietczyk A (2020). COVID-19-associated invasive Aspergillosis: data from the UK National Mycology Reference Laboratory. J. Clin. Microbiol..

[7] Howard KC, Dennis EK, Watt DS, Garneau-Tsodikova S (2020). A comprehensive overview of the medicinal chemistry of antifungal drugs: perspectives and promise. Chem. Soc. Rev..

[8] Kim JH, Park H, Seo SW (2017). In situ synthesis of silver nanoparticles on the surface of PDMS with high antibacterial activity and biosafety toward an implantable medical device. Nano Convergence.

[9] Zhao Y, Guo Q, Dai X, Wei X, Yu Y, Chen X, Li C, Cao Z, Zhang X (2019). A biomimetic non-antibiotic approach to eradicate drug-resistant infections. Adv. Mater..

[10] Wei X, Song M, Li W, Huang J, Yang G, Wang Y (2021). Multifunctional nanoplatforms co-delivering combinatorial dual-drug for eliminating cancer multidrug resistance. Theranostics.

[11] Aston WJ, Hope DE, Nowak AK, Robinson BW, Lake RA, Lesterhuis WJ (2017). A systematic investigation of the maximum tolerated dose of cytotoxic chemotherapy with and without supportive care in mice. BMC Cancer.

[12] Zhang C, Li Y, Shuai D, Shen Y, Wang D (2019). Progress and challenges in photocatalytic disinfection of waterborne Viruses: a review to fill current knowledge gaps. Chem. Eng. J..

[13] Zhao Y, Prideaux B, Baistrocchi S, Sheppard DC, Perlin DS (2019). Beyond tissue concentrations: antifungal penetration at the site of infection. Med. Mycol..

[14] Kim J, Tang JY, Gong R, Kim J, Lee JJ, Clemons KV, Chong CR, Chang KS, Fereshteh M, Gardner D (2010). Itraconazole, a commonly used antifungal that inhibits Hedgehog pathway activity and cancer growth. Cancer Cell.

[15] Seo H, Kim JY, Son HJ, Jung J, Kim MJ, Chong YP, Lee SO, Choi SH, Kim YS, Kim SH (2021). Diagnostic performance of real-time polymerase chain reaction assay on blood for invasive aspergillosis and mucormycosis. Mycoses.

[16] Joanna C, Marcin L, Ewa K, Grażyna P (2018). A nonspecific synergistic effect of biogenic silver nanoparticles and biosurfactant towards environmental bacteria and fungi. Ecotoxicology.

[17] Fang W, Sanz AB, Bartual SG, Wang B, Ferenbach AT, Farkaš V, Hurtado-Guerrero R, Arroyo J, Van Aalten DM (2019). Mechanisms of redundancy and specificity of the *Aspergillus fumigatus* Crh transglycosylases. Nat. Commun..

[18] Lai C-C, Yu W-L (2020). COVID-19 associated with pulmonary aspergillosis: a literature review. J. Microbiol. Immunol. Infect..

[19] Barai AC, Paul K, Dey A, Manna S, Roy S, Bag BG, Mukhopadhyay C (2018). Green synthesis of *Nerium oleander*-conjugated gold nanoparticles and study of its in vitro anticancer activity on MCF-7 cell lines and catalytic activity. Nano Convergence.

[20] Geißel B, Loiko V, Klugherz I, Zhu Z, Wagener N, Kurzai O, van den Hondel CA, Wagener J (2018). Azole-induced cell wall carbohydrate patches kill *Aspergillus fumigatus*. Nat. Commun..

[21] Karashima M, Sano N, Yamamoto S, Arai Y, Yamamoto K, Amano N, Ikeda Y (2017). Enhanced pulmonary absorption of poorly soluble itraconazole by micronized cocrystal dry powder formulations. Eur. J. Pharm. Biopharm..

[22] Furukawa T, van Rhijn N, Fraczek M, Gsaller F, Davies E, Carr P, Gago S, Fortune-Grant R, Rahman S, Gilsenan JM (2020). The negative cofactor 2 complex is a key regulator of drug resistance in *Aspergillus fumigatus*. Nat. Commun..

[23] Zhu S, Li L, Gu Z, Chen C, Zhao Y (2020). 15 years of small: research trends in nanosafety. Small.

[24] Al-Tayyar NA, Youssef AM, Al-Hindi RR (2020). Antimicrobial packaging efficiency of ZnO-SiO2 nanocomposites infused into PVA/CS film for enhancing the shelf life of food products. Food Packag. Shelf Life.

[25] Ma YX, Wang CY, Li YY, Li J, Wan QQ, Chen JH, Tay FR, Niu LN (2020). Considerations and caveats in combating eskape pathogens against nosocomial infections. Adv Sci.

[26] Jo YK, Choi BH, Kim CS, Cha HJ (2017). Diatom-inspired silica nanostructure coatings with controllable microroughness using an engineered mussel protein glue to accelerate bone growth on titanium-based implants. Adv. Mater..

[27] Liu H, Luan Y, Koo B, Lee EY, Joo J, Dao TNT, Zhao F, Zhong L, Yun K, Shin Y (2019). Cucurbituril-based reusable nanocomposites for efficient molecular encapsulation. ACS Sustain. Chem. Eng.

[28] Ragni R, Cicco SR, Vona D, Farinola GM (2018). Multiple routes to smart nanostructured materials from diatom microalgae: a chemical perspective. Adv. Mater..

[29] Cao D, Shu X, Zhu D, Liang S, Hasan M, Gong S (2020). Lipid-coated ZnO nanoparticles synthesis, characterization and cytotoxicity studies in cancer cell. Nano Convergence.

[30] Lee NK, Wang C-PJ, Lim J, Park W, Kwon H-K, Kim S-N, Kim T-H, Park CG (2021). Impact of the conjugation of antibodies to the surfaces of polymer nanoparticles on the immune cell targeting abilities. Nano Convergence.

[31] Qiao Z, Seo H, Liu H, Cha H-H, Kim JY, Kim S-H, Shin Y (2021). Simple and sensitive diagnosis of invasive aspergillosis using triphasic DE−ZnO−APDMS microparticle composite. Sensors Actuators B: Chemical.

[32] Dananjaya S, Kumar RS, Yang M, Nikapitiya C, Lee J, De Zoysa M (2018). Synthesis, characterization of ZnO-chitosan nanocomposites and evaluation of its antifungal activity against pathogenic *Candida albicans*. Int. J. Biol. Macromol..

[33] Lee J, Lee HA, Shin M, Juang LJ, Kastrup CJ, Go GM, Lee H (2020). Diatom frustule silica exhibits superhydrophilicity and superhemophilicity. ACS Nano.

[34] Yang B, Chen Y, Shi J (2019). Reactive oxygen species (ROS)-based nanomedicine. Chem. Rev..

[35] Billmyre RB, Clancey SA, Li LX, Doering TL, Heitman J (2020). 5-fluorocytosine resistance is associated with hypermutation and alterations in capsule biosynthesis in Cryptococcus. Nat. Commun..

[36] Bhadra, D. P.; Dutta, S. B.; Bhattyacharya, D.; Mukherjee, S., Comparative Study of the Zno and Zno Coated with Sio2 As Potential Antimicrobial and Anticancer Drugs. Bioscience Biotechnology Research Communications (Official Journal of Society for Science À Nature) **2019,** *12*.

[37] Mirouze N, Ferret C, Cornilleau C, Carballido-López R (2018). Antibiotic sensitivity reveals that wall teichoic acids mediate DNA binding during competence in *Bacillus subtilis*. Nat. Commun..

[38] Kang J, Dang V, Li H, Moon S, Li P, Kim Y, Kim C, Choi H, Liu Z, Lee H (2016). InGaN-based photoanode with ZnO nanowires for water splitting. Nano Convergence.

[39] Efthimiou I, Georgiou Y, Vlastos D, Dailianis S, Deligiannakis Y (2020). Assessing the cyto-genotoxic potential of model zinc oxide nanoparticles in the presence of humic-acid-like-polycondensate (HALP) and the leonardite HA (LHA). Sci. Total Environ..

[40] Ansari AA, Hasan TN, Syed NA, Labis JP, Parchur A, Shafi G, Alshatwi AA (2013). In-vitro cyto-toxicity, geno-toxicity, and bio-imaging evaluation of one-pot synthesized luminescent functionalized mesoporous SiO2@ Eu (OH) 3 core-shell microspheres. Nanomed. Nanotechnol. Biol. Med..

[41] Molahasani N (2018). Investigation of cytotoxicity properties of zinc oxide nanoparticles in spherical and rod shaped on leukemia cells. Int. J. Bio-Inorg. Hybr. Nanomater.

