# Supplementary material for: Diatomaceous earth/zinc oxide micro-composite assisted antibiotics in fungal therapy
Source: Nano Converg. 2021 Oct 25;8:32. doi: 10.1186/s40580-021-00283-6 (PMC8542915; doi:10.1186/s40580-021-00283-6)
Supplement: Supplementary file 1 — Additional file 1. Additional Figures and Table. [file 40580_2021_283_MOESM1_ESM.docx]

**Supplementary**

**Diatomaceous Earth/Zinc Oxide Micro-Composite Assisted Antibiotics in Fungal Therapy**

Huifang Liu^1,†^, Zhen Qiao^1,†^, Yoon Ok Jang^1^, Myoung Gyu Kim^1^, Qingshuang Zou^1^, Hyo Joo Lee^1^, Bonhan Koo^1^, Sung-Han Kim^2^, Kyusik Yun^3^, Hyun-Soo Kim^4^ and Yong Shin^1,*^

^1^Department of Biotechnology, College of Life Science and Biotechnology, Yonsei University, Korea

^2^Department of Infectious Disease, Asan Medical Center, University of Ulsan College of Medicine, 88 Olympicro-43gil, Songpa-gu, Seoul 05505, Republic of Korea

^3^Department of Bionanotechnology, Gachon University, Gyeonggi-do 13120, Republic of Korea

^4^Infusiontech company, Anyangsi, Gyeonggi-do, Republic of Korea

†These authors (H.L & Z.Q) contributed equally to this work.

*Correspondence: shinyongno1@yonsei.ac.kr; Tel.: +82-2-2123-2885

Figure S1 Characterization of the comparable ZnO nanomaterials.

Figure S2. Figure of fungi culture with pure ZnO nanomateirals. (Amount control)

Figure S3 Energy-dispersive X-ray spectroscopy of the DE-ZnO in different modification ratio.

Figure S4. Figure of fungi culture with DE-ZnO nanocomposites. (Amount control)

Figure S5. Study the toxicity of DE-ZnO.

Table S1. Biocompatibility of DE-ZnO composites effects on ALT and AST in mice.

**
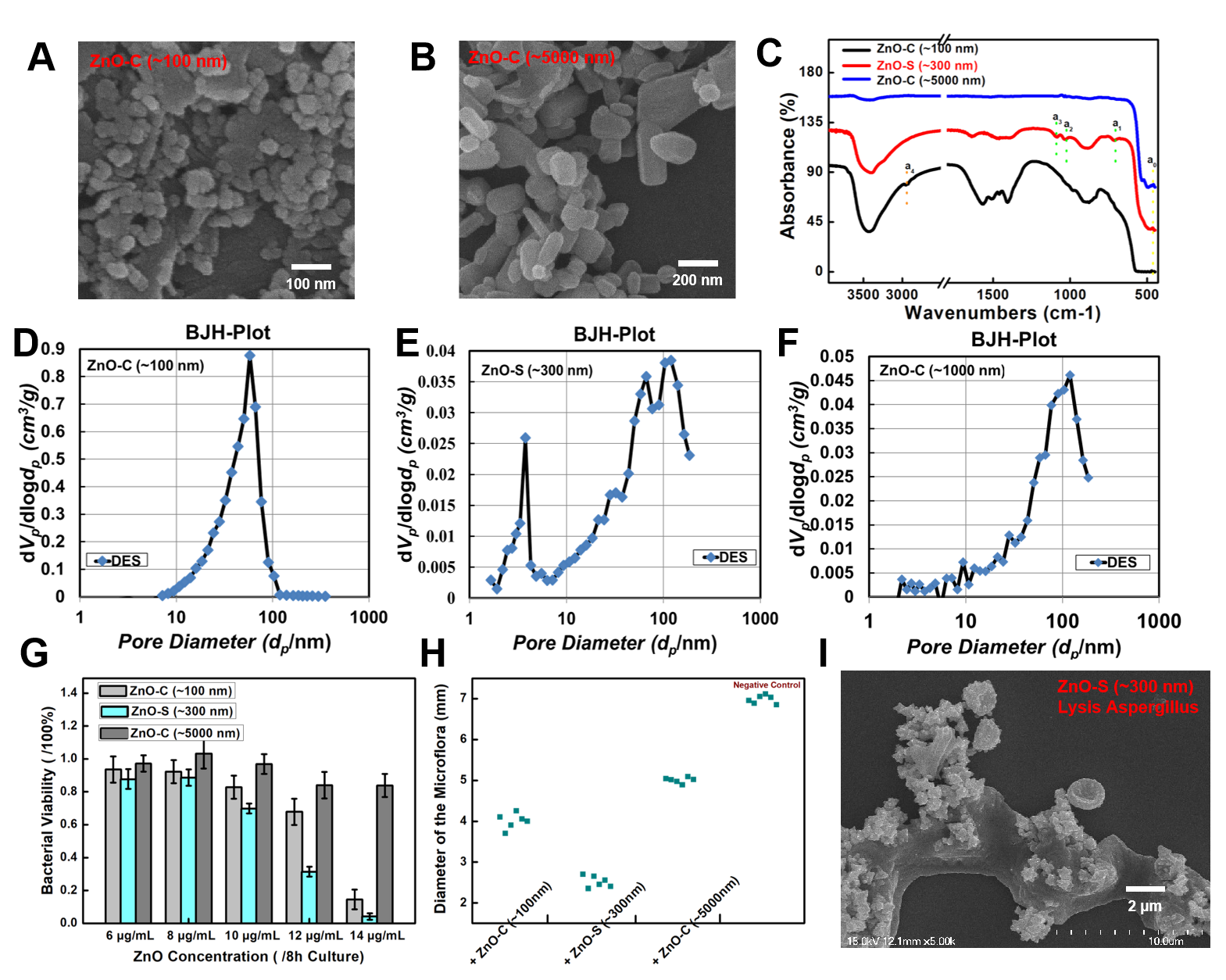
**

**Figure S1 Characterization of the comparable ZnO nanomaterials.**

**a** SEM images of commercial nanoparticles ZnO-C (~100 nm). **b** SEM images of commercial nanoparticles ZnO-C (~5000 nm). FTIR spectra of the ZnO nanomaterials; **c** Zeta potential of the ZnO nanomaterials. **d~f** Barrett-Joyner-Halenda (BJH) pore size distribution plots of ZnO-C (~100 nm), ZnO-S (~300 nm) and ZnO-C (~5000 nm). **g** Antibacterial efficiency of the ZnO nanomaterials against Gram-negative bacteria at different concentrations (µg/mL) tested on E. coli after 12 h incubation. Data presented are mean ± SE (n = 3). **h** Antifungal efficiency of the ZnO nanomaterials shown by the diameter of the microflora of *A. fumigatus* at a concentration of 10 µg/mL. **i** SEM image of ZnO-S (~300 nm) lysis of *A. fumigatus.*


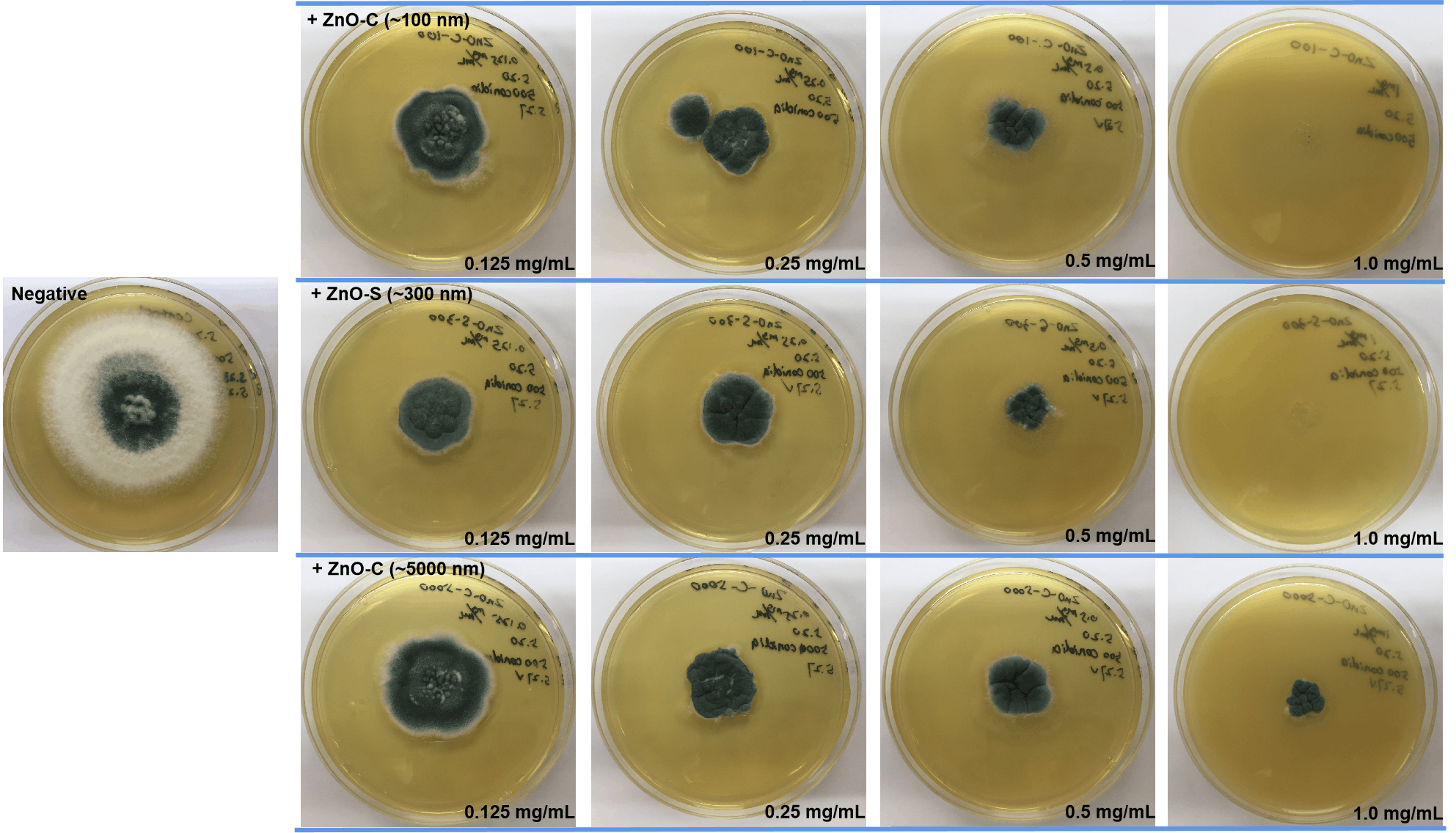


**Figure S2. Colony figure of fungi culture with pure ZnO nanomaterials. (Amount control)**

**
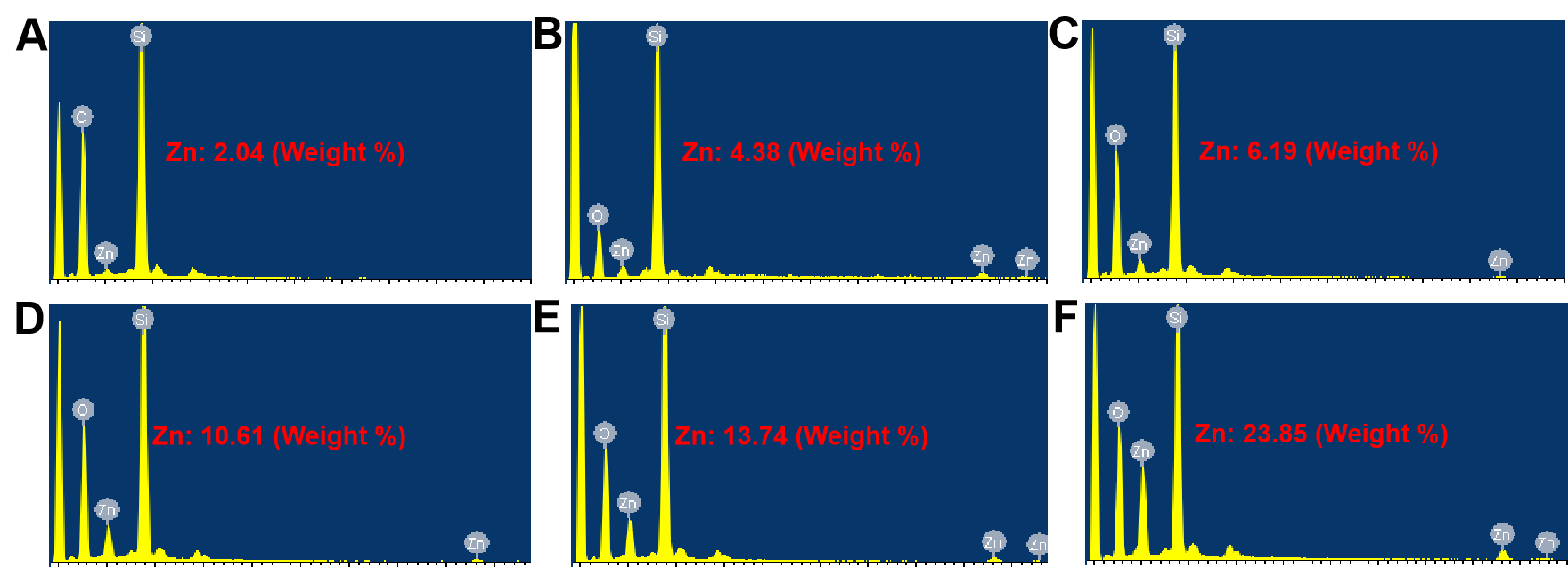
 Figure S3 Energy-dispersive X-ray spectroscopy of the DE-ZnO in different modification ratio.**

ZnO : DE= 0.25:1; 0.5:1; 1:1; 2:1; 3:1; 4:1) The percentage of ZnO are 2.04%, 4.38%, 6.19%, 10.61%, 13.74% and 23.85%


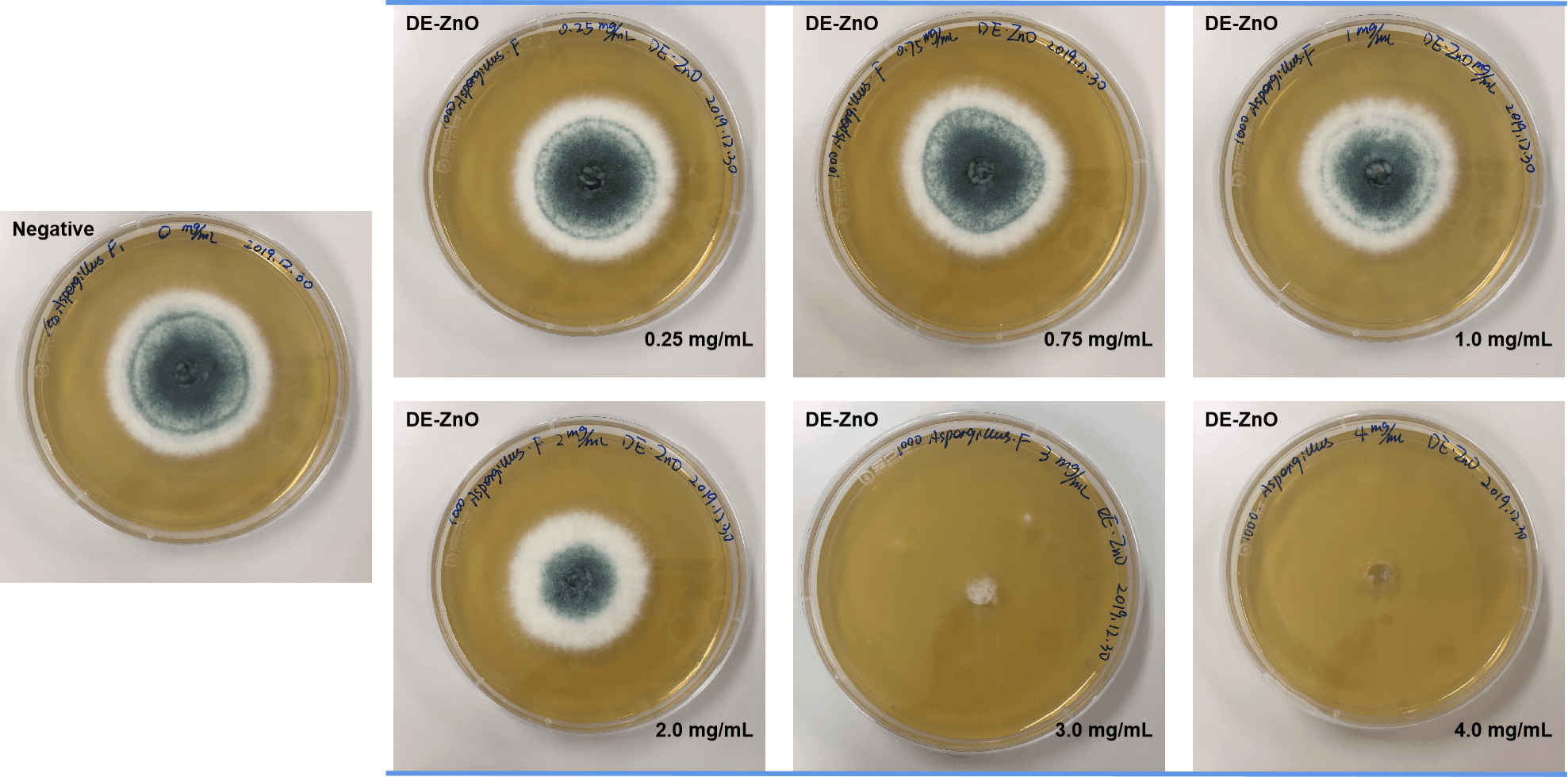


**Figure S4. Colony figure of fungi culture with DE-ZnO nanocomposites. (Amount control)**

**
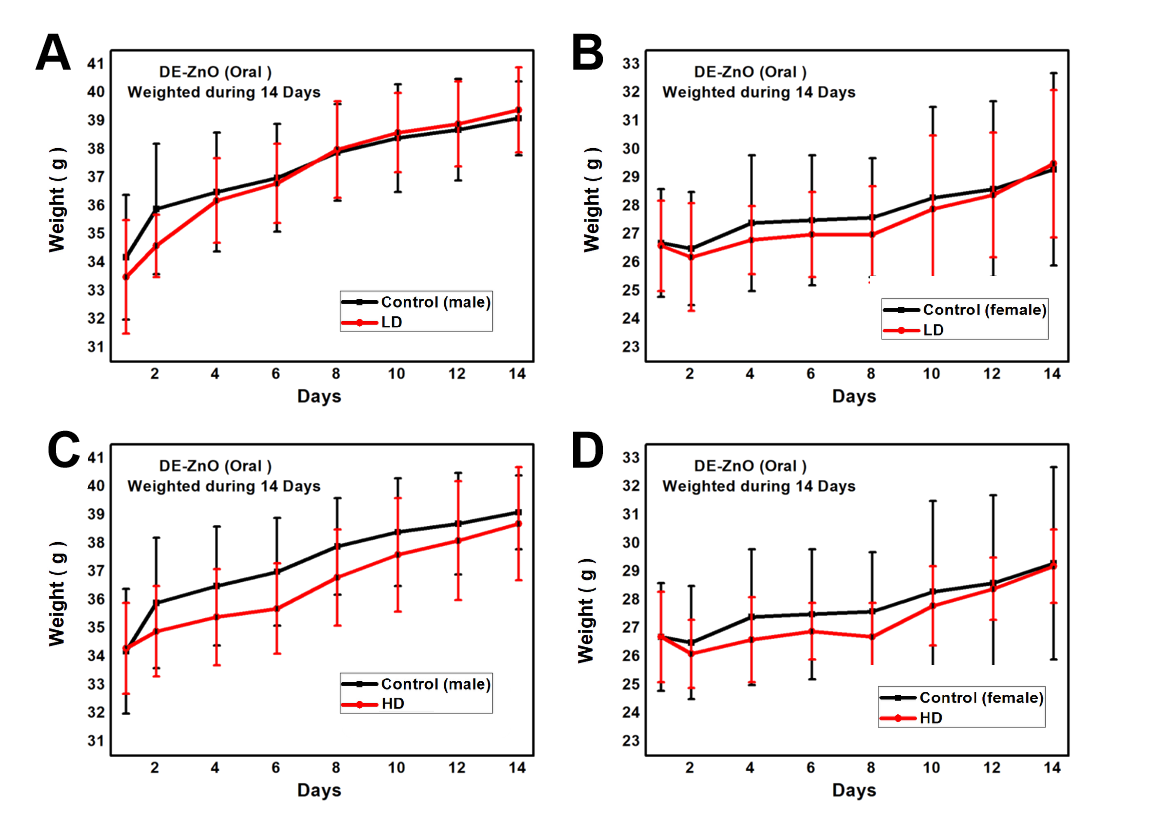
**

**Figure S5. Study the toxicity of DE-ZnO**

**a~d** after oral take the DE-ZnO, body weight gains of the mice have been traced in 14 days growth; male and female individual test. Both LD-DE-ZnO and HD-DE-ZnO have been studied, and the data values are mean ± SE. Abbreviations: LD, low dosage; HD, high dosage.

**Table S1. Biocompatibility of DE-ZnO3 composites effects on ALT and AST in mice**

| Groups | Injection Mode | AST (U/L) | | ALT (U/L) | |
| --- | --- | --- | --- | --- | --- |
|  |  | Male (n=4) | Female (n=4) | Male (n=4) | Female (n=4) |
| Control | Oral | 75.4±17 | 54.5±3.1 | 43.2±6.2 | 32.1±4.9 |
| LD-DE-ZnO |  | 69.4±14.7 | 59.8±7.3 | 32.3±4.0 | 26.6±4.9 |
| HD-DE-ZnO |  | 70.3±24.9 | 74.8±14.3 | 40.8±17.8 | 39.1±2.3 |

Each data values are mean ± SE (n = 4 mice per group).

Abbreviations: ALT, alanine aminotransferase; AST, aspartate aminotransferase; LD, low dosage; HD, high dosage
